# Evaluation of the Ionospheric Corrections Generated by Smartphones with Different Real-Time Products

**DOI:** 10.3390/s26061795

**Published:** 2026-03-12

**Authors:** Yan Zhang, Yang Jiang, Yang Gao

**Affiliations:** Department of Geomatics Engineering, University of Calgary, Calgary, AB T2N1N4, Canada; yang.jiang1@ucalgary.ca (Y.J.); ygao@ucalgary.ca (Y.G.)

**Keywords:** smartphone positioning, PPP-RTK, CNES, PPP-B2b, time-differenced carrier phase

## Abstract

Ionospheric delay is a dominant error source in global navigation satellite systems (GNSSs). Conventional ionospheric estimation relies on dense networks of expensive geodetic receivers, limiting accessibility and coverage. With the widespread availability of multi-frequency, multi-constellation smartphones capable of carrier-phase tracking, this study investigates smartphone-based ionospheric estimation. Using a single-reference Precise Point Positioning Real-Time Kinematic (PPP-RTK) framework, ionospheric delays are estimated from smartphone data and evaluated using real-time correction products from BeiDou PPP-B2b and Centre National d’Études Spatiales (CNES). Quality control is performed via solution separation testing with time-differenced carrier phase and time-differenced pseudorange. Field experiments with two Google smartphones and a geodetic receiver demonstrate that the estimated slant ionospheric accuracy is comparable to geodetic receivers within the meter level under both static and kinematic scenarios. Additionally, the horizontal positioning performance demonstrates that the positioning performance of the user smartphone with ionospheric corrections broadcast from the base smartphone is significantly improved, with 74.7% and 54.9% for CNES and PPP-B2b products compared with the conventional PPP solution. Furthermore, a comparison between ionospheric corrections generated from the smartphone and those obtained from the geodetic receiver reveals that the positioning performance of the user smartphone becomes comparable after convergence.

## 1. Introduction

The ionospheric delay is a major source of error affecting the positioning, navigation, and timing (PNT) performance of global navigation satellite systems (GNSSs), particularly for applications such as autonomous driving, precision agriculture, disaster monitoring, and geoscientific surveying [1,2,3]. The ionospheric delay, caused by the dispersive nature of the ionized upper atmosphere, introduces frequency-dependent range errors that can reach up to 15 m during the daytime and 3 m at night and may increase to 40 m vertically or even 100 m along the line of sight during periods of high solar activity [4,5,6]. Accurate estimation and mitigation of ionospheric effects are therefore essential for high-precision techniques such as Precise Point Positioning Real-Time Kinematic (PPP-RTK) [7,8].

Traditionally, ionospheric corrections for PPP-RTK are generated using networks of permanently deployed, high-end geodetic GNSS reference stations. These networks provide high-quality observations with low noise and high sampling rates, enabling the estimation of slant ionospheric delays or ionospheric corrections at the user location through spatial interpolation or state-space representation (SSR) techniques [9,10,11]. While such approaches have proven effective for professional and scientific applications, they suffer from several inherent limitations. First, the establishment and maintenance of dense geodetic networks are costly, restricting their deployment to regions with sufficient infrastructure and economic resources [12]. Second, the spatial sparsity and limited temporal resolution of reference networks can lead to degraded ionospheric corrections in areas with poor coverage, highly dynamic ionospheric conditions, or localized disturbances, such as urban canyons and low-latitude regions during periods of high solar activity [13,14]. These limitations significantly constrain the scalability of conventional PPP-RTK services for mass-market and consumer-grade applications.

In recent years, the rapid proliferation of low-cost GNSS receivers has created new opportunities for ionospheric modeling and precise positioning. In particular, modern smartphones are now equipped with multi-frequency and multi-constellation GNSS chipsets capable of providing raw pseudorange and carrier-phase observations [15,16,17,18]. Since the release of the Android GNSS raw measurement interface, a growing body of research has demonstrated the feasibility of using smartphones for precise positioning, including PPP and RTK, under both static and kinematic conditions [19,20,21]. Although smartphone GNSS observations are generally noisier than those from geodetic-grade receivers, their global density and continuous availability offer a compelling alternative for large-scale, low-cost ionospheric sensing [22,23].

While the potential of smartphone GNSSs for high-precision positioning is promised, the accurate estimation of ionospheric delays for PPP-RTK using smartphones has received little attention in existing studies, which primarily concentrates on: (1) smartphone positioning performance under static or kinematic conditions [24,25], with limited exploration of ionospheric delay estimation accuracy; (2) ionospheric correction using low-cost dedicated receivers (ublox F9P) [26,27], which lack the accessibility of smartphone hardware; or (3) single-frequency ionospheric modeling [28], which fails to leverage the multi-frequency capabilities of modern smartphones. Moreover, few studies have systematically compared the performance of ionospheric corrections derived from smartphones using different real-time satellite orbit/clock products (e.g., satellite-based vs. ground-based), leaving uncertainty about the adaptability of smartphone-derived ionospheric delays to diverse correction sources for PPP-RTK positioning. This research gap motivates the present study to develop and validate smartphone-based ionospheric delay estimation methods for PPP-RTK in real-time scenarios.

This study aims to develop and evaluate methods for estimating ionospheric delays by smartphones and to assess their suitability for PPP-RTK applications. Specifically, ionospheric delay estimates are generated using a single reference PPP-RTK approach based on uncombined observations. Two independent real-time satellite orbit, clock, and code bias products are considered: the satellite-based BeiDou PPP-B2b service and the ground-based International GNSS Services (IGS) Real-Time Services (RTS) products provided by the Centre National d’Études Spatiales (CNES). Quality control measures based on solution separation testing are applied to assess the consistency and reliability of the raw smartphone observations. The ionospheric delays estimated from smartphone observations are then compared with those obtained using a high-end geodetic GNSS receiver to evaluate their accuracy. Finally, the derived ionospheric corrections are applied to a single-reference PPP-RTK positioning solution to assess smartphone positioning performance under kinematic conditions. The goal is to evaluate the accuracy of ionospheric delay estimates derived from smartphone GNSS data and to assess their impact on the positioning performance of PPP-RTK.

## 2. Methods

In this section, the quality control methods applied in smartphone GNSS data processing are first introduced, followed by an evaluation of ionospheric corrections using the uncombined PPP approach.

### 2.1. Quality Control Methods for Smartphone Observations

The GNSS observations obtained from smartphones are highly susceptible to environmental interference, resulting in frequent cycle slips and a high noise level of pseudorange measurements. First, the solution separation (SS) testing method with timed-differenced carrier phase (TDCP) is applied to detect the cycle slips, and then the outlier detection of pseudorange is used to deal with the displacement consistency check between the TDCP and time-differenced pseudorange (TDPR).

By differencing the measurements from two consecutive GNSS epochs in the carrier-phase measurement equation, the TDCP observation equation reads [29]

(1) $$\Delta L_{i}^{s}=\Delta \rho_{i}^{s}+c\left(\Delta {dt}_{r}-{\Delta dT}^{s}\right)+{\Delta T}_{s}-\gamma^{i}\Delta I_{r,1}+\lambda \Delta N_{i}^{s}+\varepsilon_{{\Delta L}_{i}}$$

where $\Delta L_{r}^{s}$ represents the epoch-differenced carrier-phase measurement, $\Delta \rho_{r}^{s}$ represents the displacement of the user between two epochs, $\Delta {dt}_{r}$ and ${\Delta dT}^{s}$ denote the epoch-differenced clock offsets, ${\Delta T}_{r}^{s}$ and $\Delta I_{r,1}$ are the differenced atmospheric errors, which can be ignored with 1 s sampling interval, $\Delta N_{i}^{s}$ means the epoch-differenced ambiguity, which reflects the possible existence of cycle slips, c is the speed of light in a vacuum, i is the frequency number, $\gamma^{i}$ is the frequency amplification factor, λ is the signal wavelength, and $\epsilon_{r,\Delta L}^{s}$ represents the differenced measurement noise.

Therefore, after obtaining the matrix form of the TDCP model, an SS testing technique is used for cycle slip detection [30]. We suppose that the ${\hat{X}}_{0}$ and ${\hat{X}}_{k}$ are the least-squares estimation of whole TDCP measurements and the solution with exclusion of the observable element $\Delta L_{i,k}^{s}$. The difference between ${\hat{X}}_{0}$ and ${\hat{X}}_{k}$ is given below:

(2) $$\Delta_{k}^{SS}={\left|{\hat{X}}_{k}-{\hat{X}}_{0}\right|}_{m\times 1}$$

(3) $${∆}_{max}^{SS}={max}_{k=1}^{m}({∆}_{k}^{SS})$$

Then, the SS threshold $T_{d}$ is calculated, which is given as

(4) $$T_{d}=\sigma Q^{-1}\left(\frac{P_{FA}}{4n}\right)$$

(5) $$Q\left(u\right)=\frac{1}{\sqrt{2\pi }}\int_{u}^{+\infty }e^{-\frac{t^{2}}{2}dt}$$

where σ is the standard deviation on ${\hat{X}}_{k}$, Q is the tail probability of a standard normal distribution with a mean of zero, $Q^{-1}$ is the inverse of Q, $Q^{-1}(p)$ denotes $\left(1-p\right)$, the quantile of a standard normal distribution with zero mean and unit variance, $P_{FA}$ denotes the integrity requirement of false-alarm probability, m is the number of valid TDCP observations, and n is the number of unknown parameters, which includes $[x_{TDCP}\;y_{TDCP}\;z_{TDCP}\;c\Delta dt_{r}{]}^{T}$, representing the receiver position and clock bias. Comparing ${∆}_{k}^{SS}$ with $T_{d}$, if $\Delta_{k}^{SS}>T_{d}$, the SS testing fails, meaning that a fault exists, and vice versa. Then, we remove the observation with the largest ${∆}_{k}^{SS}$, denoted as ${∆}_{max}^{SS}$.

Due to the high precision of carrier-phase measurements, the TDCP observation $\Delta L_{i}^{s}$ provides an accurate estimate of the short-term geometric range variation. By forming the difference between pseudorange and carrier-phase epoch differences, a consistency residual can be constructed as

(6) $$r_{i}^{s}=\Delta P_{i}^{s}-\Delta L_{i}^{s}=2\Delta I_{r,1}+\eta_{i}^{s}$$

where $\Delta P_{i}^{s}$ and $\Delta L_{i}^{s}$ are TDCP and TDPR measurements and $\eta_{i}^{s}$ includes measurement noise and unmodeled effects. As the cycle slips have been detected there, the carrier-phase measurements are recognized as consistent. Thus, the TDCP and TDPR equations are given as

(7) $$\Delta L_{i}^{s}=\Delta \rho_{i}^{s}+c\left(\Delta {dt}_{r}-{\Delta dT}^{s}\right)+{\Delta T}_{s}-\gamma^{i}\Delta I_{r,1}+\varepsilon_{{\Delta L}_{i}}$$

(8) $$\Delta P_{i}^{s}=\Delta \rho_{i}^{s}+c\left(\Delta {dt}_{r}-{\Delta dT}^{s}\right)+{\Delta T}_{s}+\gamma^{i}\Delta I_{r,1}+\varepsilon_{{\Delta P}_{i}}$$

For short sampling intervals, the ionospheric variation term is typically small and slowly varying. Consequently, the residual mainly reflects pseudorange measurement abnormalities. To perform statistical detection, the residual is normalized as

(9) $$z_{i}^{s}=r_{i}^{s}/\sigma_{r,i}^{s}$$

where $\sigma_{r,i}^{s}$ denotes the standard deviation of the TDCP consistency residual, obtained from the error propagation of pseudorange and carrier-phase noise models. Assuming Gaussian behavior under nominal conditions, the test statistic approximately follows a standard normal distribution $z_{i}^{s}\sim N(0,\;1)$.

A pseudorange gross error is declared when $\left|z_{i}^{s}\right|>\kappa$, and κ is the detection threshold determined according to a predefined probability of false alarm (PFA) with $10^{-5}$.

### 2.2. Uncombined PPP Model to Estimate Ionospheric Corrections

For receiver r, satellite s and frequency i, the undifferenced pseudorange $P_{r,i}^{s}$ and carrier phase $L_{r,i}^{s}$ are modeled as

(10) $$P_{r,i}^{s}=\rho_{i}^{s}+c\cdot \left(dt_{r}-dT^{s}\right)+d_{orb}+T_{s}+\gamma^{i}\cdot I_{r,1}+b_{r,i}^{P}-b_{i}^{P,s}+\varepsilon_{P_{i}}$$

(11) $$L_{r,i}^{s}=\rho_{i}^{s}+c\cdot \left(dt_{r}-dT^{s}\right)+d_{orb}+T_{S}-\gamma^{i}\cdot I_{r,1}+\lambda_{i}N_{r,i}^{s}+b_{r,i}^{L}-b_{i}^{L,s}+\varepsilon_{L_{i}}$$

where $dT^{s}$ represents satellite clock bias, $dt_{r}$ represents receiver clock biases, and $d_{orb}$ represents the orbital error. In this study, these three error components are corrected using real-time correction products from BDS PPP-B2b and CNES. $b^{P}$ and $b^{L}$ represent code/phase hardware biases (receiver and satellite).

The ionosphere is dispersive and can be parameterized by a single slant ionospheric delay at the first frequency (L1) with frequency scaling factor $\gamma^{i}$ ($\gamma^{i}=f_{i}/f_{j}$, i and j are frequency numbers). Accordingly, UC PPP estimates one ionospheric parameter per satellite (per epoch, typically as a random walk), while mapping it to each frequency. After applying standard deterministic corrections such as antenna phase center variations, phase wind-up, relativistic effects, tides, etc. [31], a typical UC PPP state vector for ionosphere estimation is

(12) $$X=[p_{r}^{s},dt_{r},I_{r,1},T_{S},N_{r,i}^{s}]$$

where $p_{r}^{s}$ stands for the receiver’s coordinates. In this study, we want to evaluate the accuracy of ionospheric corrections on the user side; due to the limitation of the ionospheric corrections generated from a single reference smartphone, traditional methods to apply ionospheric corrections from the base to the user [32,33] are not applicable. In this study, the correction compensations of the ionosphere can be described by empirical models [3]:

(13) $$I_{R,1}=I_{B,1}-I_{B,M}+I_{R,M}$$

(14) $$C_{I_{R,1}}=C_{I_{B,1}}+a({\left({lat}_{B}-{lat}_{R}\right)}^{2}+{\left({lon}_{B}-{lon}_{R}\right)}^{2})/{(\sin\theta )}^{2}$$

$I_{R,1}$ denotes the compensation of ionospheric delay at the user side; $I_{B,1}$ represents the estimated ionospheric delay at the base; $I_{B,M}$ is the modeled ionospheric delay at the base; and $I_{R,M}$ is the modeled ionospheric delay at the rover. $C_{I_{R,1}}$ denotes the ionospheric observation noise; $C_{I_{B,1}}$ is the variance–covariance of ionospheric observation at the base; ${lat}_{B}$ and ${lat}_{R}$ represent the latitude of the base and rover; ${lon}_{B}$ and ${lon}_{R}$ are the longitude of the base and rover; θ is the elevation angle of the satellite; L is the baseline length; and a is an empirical coefficient set to 100 in this paper, following a previous study [34]. For the receiver hardware bias terms in the pseudorange observations, they will be absorbed by the slant ionospheric parameters and the receiver clock bias. This parameter in ionospheric estimation represents the hardware bias of a receiver, which is stable over a short period and thus can be modeled by the random walk process with a small spectral density (taken as 0.0001 m^2^/s in this paper) [34].

To evaluate the positioning performance when ionospheric corrections are applied on the user side, the ionospheric constraint parameters are first estimated using approximate initial values. During the early stages of PPP convergence, these parameters generally exhibit large prior covariances. Consequently, the external augmentation corrections are given more weight in the beginning to accelerate the convergence of atmospheric parameter estimation.

**Figure 1:**
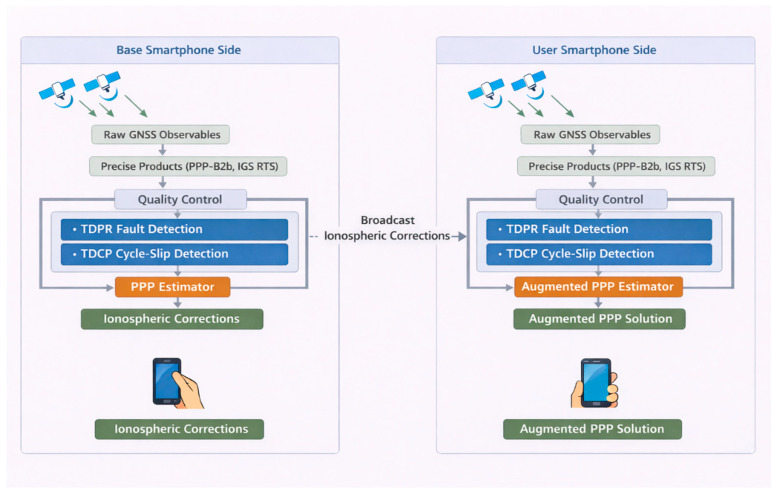
Architecture of smartphone-based ionospheric correction estimation and PPP-RTK augmentation. Three types of arrows are used in the figure: (1) arrows from the satellites denote the transmitted satellite signals; (2) arrows within the main processing framework indicate the flow of data and processing steps between different modules; and (3) arrows between the base and the user represent the broadcasting of ionospheric corrections from the base side to the user side.

Figure 1 presents the architecture of smartphone-based ionospheric corrections estimation and application to PPP-RTK. The base smartphone processes GNSS data with real-time precise products to estimate and broadcast ionospheric corrections. The user smartphone combines these corrections with its own GNSS observations to achieve improved PPP performance. Quality control methods, including TDPR fault detection and SS-based TDCP cycle-slip detection, are applied on both sides to ensure robust and accurate positioning.

In this study, the PPP-RTK framework is implemented without integer ambiguity resolution, as the primary objective is to evaluate the accuracy and positioning impact of smartphone-derived ionospheric correction performance. On the user side, only ionospheric corrections broadcast from the service side are applied, while no phase bias information is used; therefore, both the conventional PPP solution and the proposed ionosphere-augmented solution operate in float ambiguity mode.

## 3. Experimental Results and Analysis

This section for this study introduces static and kinematic experiment setups, results and analysis. The observation collected and used in this study is received by the smartphone without external antennas or signal repeaters.

### 3.1. Experiment Setups

To rigorously evaluate the accuracy of ionospheric corrections generated from a single smartphone, as well as the positioning performance with the augmentation of these corrections, a real-world field experiment was designed and conducted on 7 April 2024, in Calgary (51.0447° N, 114.0719° W), Canada. The experimental setup included two Android smartphones configured as a base station and a user station, respectively, along with a high-end Trimble NetR9 receiver. The Trimble receiver was installed in an open-sky environment at the top of the Engineering Building at the University of Calgary, and the ionospheric corrections derived from this geodetic-grade receiver were used as reference values for comparison with those generated by the base smartphone.

**Figure 2:**
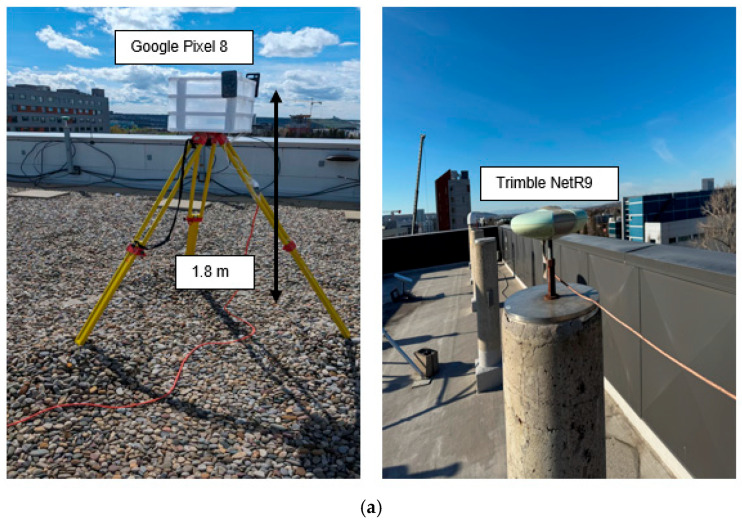

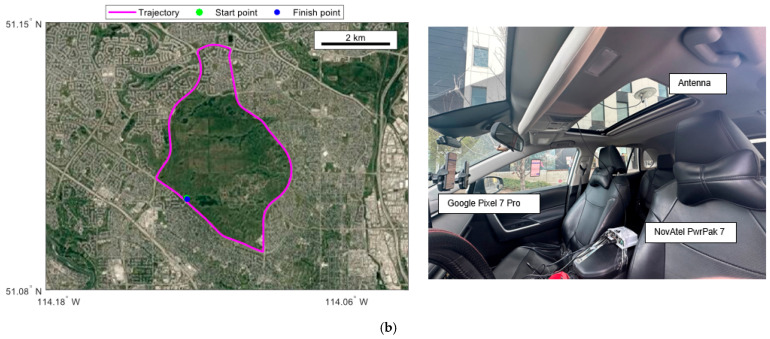
Experiment setups of static and kinematic experiments. (**a**) In the static experiment, a Google Pixel 8 smartphone is placed on the tripod facing north, and the height of the tripod is approximately 1.8 m from the ground. (**b**) In the kinematic experiment, a Google Pixel 7 Pro smartphone is placed inside a vehicle, and a NovAtel PwrPak 7 system is used as a reference.

The two smartphones used in the experiment were a Google Pixel 8 and a Google Pixel 7 Pro. The Pixel 8 was deployed as the base station and placed in a stationary open-sky environment on the rooftop of the Calgary Centre for Innovative Technology (CCIT) Building. On the user side, the Pixel 7 Pro was mounted inside a vehicle to simulate typical kinematic positioning conditions. The vehicle followed a test route that predominantly covered open-sky environments while still reflecting realistic operational scenarios encountered in suburban areas. The spatial layout and coverage of the user trajectory are illustrated in Figure 2, confirming that the route was largely under open-sky conditions. This configuration helps isolate the impact of the proposed method from environmental disturbances such as signal blockage and multipath.

Data collection lasted approximately 30 min, during which raw GNSS measurements were continuously logged on both smartphones using the GNSS Logger App (version 3.0.6.4). The baseline distance between the base and user device was approximately 5.3 km, which is suitable for evaluating ionospheric-assisted positioning, as ionospheric delays at both ends remain highly correlated over this distance.

To generate a high-precision reference trajectory, a NovAtel PwrPak7 system (Calgary, AB, Canada)—integrated with a high-grade GNSS receiver and an inertial measurement unit (IMU)—was employed. Post-processing was performed using Inertial Explorer software (version 9.0), including lever-arm calibration between the GNSS antenna and the IMU. This reference solution served as the ground truth for assessing the positioning accuracy of the user-side smartphone.

**Figure 3:**
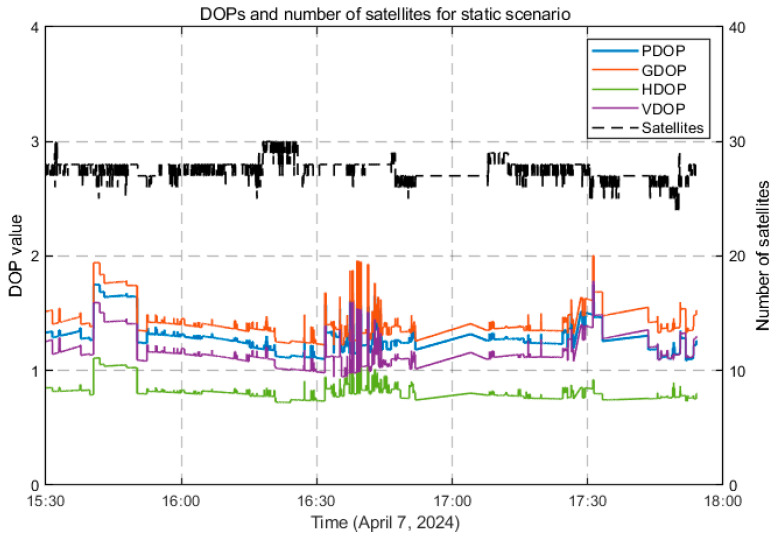
DOPs and number of satellites for Google Pixel 8 in static scenarios.

**Figure 4:**
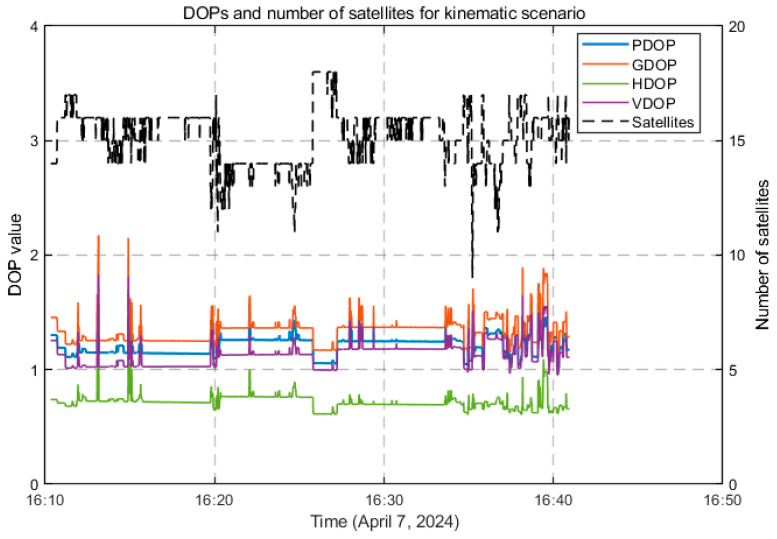
DOPs and number of satellites for Google Pixel 7 Pro in kinematic scenarios.

During the experiment, the number of available satellites on the user side varied between 24 and 30 for static scenarios and 9 and 18 for kinematic scenarios, depending on environmental conditions and satellite geometry, as shown in Figure 3 and Figure 4. The average Position Dilution of Precision (PDOP) for the static and kinematic scenarios was 1.214 and 1.289, respectively. The corresponding Horizontal Dilution of Precision (HDOP), Vertical Dilution of Precision (VDOP), and Geometry Dilution of Precision (GDOP) values were 0.716 and 0.818, 1.128 and 1.166, and 1.337 and 1.425, indicating generally favorable satellite geometry conducive to accurate positioning.

During the experiment period, the geomagnetic indices of the disturbance storm (Dst) index remained above −20 nT, which can be referred from the International Service of Geomagnetic Indices (ISGI, https://www.gfz-potsdam.de, accessed on 31 May 2024), indicating quiet geomagnetic conditions.

Table 1 summarizes the main configuration and key processing parameters used for estimating ionospheric corrections from smartphone GNSS observations, including observation screening thresholds, supported constellations, stochastic models, and Kalman filter settings. The configurations for stochastic models refer to an existing study [21]. The selected settings are consistent with commonly used practices in PPP/PPP-RTK studies and are designed to ensure reliable and reproducible positioning results. The only configuration difference between the conventional PPP and the proposed method in this study lies in the ionospheric treatment: the conventional PPP is implemented without external ionospheric constraints, whereas the proposed method incorporates ionospheric correction constraints estimated from the base smartphone. For the user-side smartphone GNSS processing, no external tropospheric products were applied; instead, the tropospheric wet delay was directly estimated using a random walk model.

**Figure 5:**
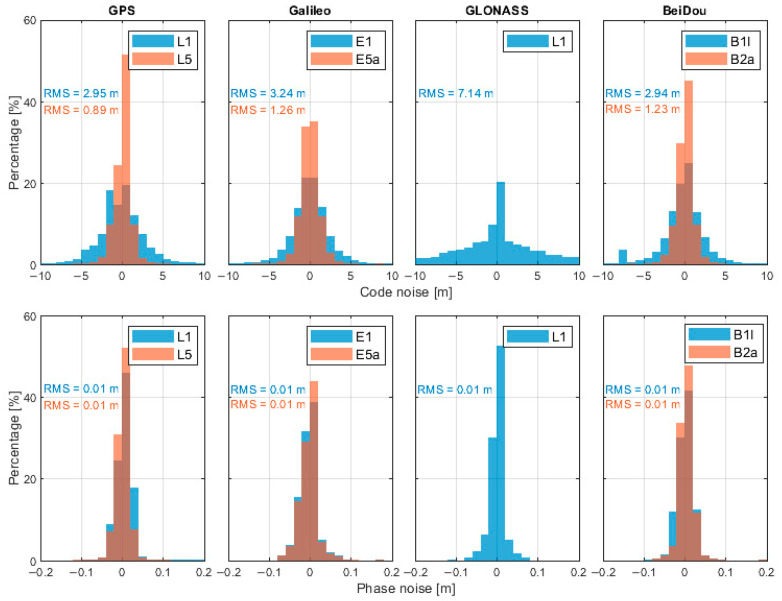
Pseudorange and phase noise for Google Pixel 7 Pro. The regions where the two colors overlap indicate the performance of another dataset.

Figure 5 illustrates the noise characteristics of L1 and L5 observations of the Google Pixel 7 Pro in kinematic scenarios. The results indicate that the code noise level on the L5 frequency is noticeably smaller than that on L1, which can be attributed to the higher chip rate and improved signal structure of the L5 signal. In contrast, the carrier-phase noise levels of L1 and L5 are generally comparable, suggesting that phase observations are less sensitive to signal modulation differences.

### 3.2. Accuracy Analysis of Smartphone-Derived Ionospheric Corrections

To evaluate the accuracy of smartphone-derived ionospheric corrections, both static and kinematic user scenarios were considered by comparing the smartphone estimates with those obtained from a geodetic-grade receiver.

In the static scenario, a Google Pixel 8 smartphone was used as both the base and the user device, while a Trimble NetR9 receiver located approximately 120 m away served as the reference. Given the limited spatial variability of the ionosphere over such a short baseline, the ionospheric corrections estimated from the Pixel 8 were compared with those derived from the Trimble NetR9 after spatially mapping both estimates to the Pixel 8 location.

In the kinematic scenario, the Pixel 8 smartphone acted as the base side, while a Google Pixel 7 Pro served as the user side. The ionospheric corrections estimated by the Pixel 8 and the Trimble NetR9 were each mapped to the trajectory of the Pixel 7 Pro, enabling a quantitative comparison of the smartphone-based and geodetic-receiver-based ionospheric corrections under dynamic conditions.

In this study, ionospheric corrections are expressed in total electron content units (TECU), which can be converted to meters as follows:

(15) $$I_{f}\;[m]=40.3\times \frac{STEC\;\left[\mathrm{TECU}\right]\times 10^{16}}{f^{2}},$$

where f is the frequency in Hz and STEC is the slant total electron content expressed in TECU, with $1\;\mathrm{TECU}=10^{16}$ electrons/m^2^.

**Figure 6:**
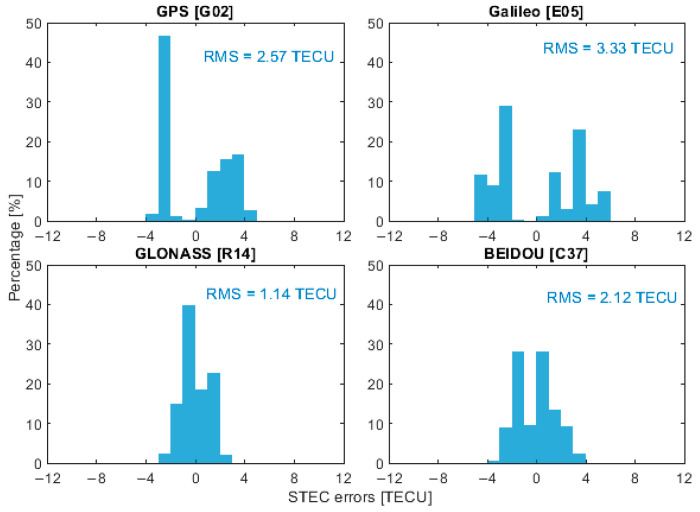
Slant ionospheric error distribution of selected satellites with CNES products in static scenarios.

**Figure 7:**
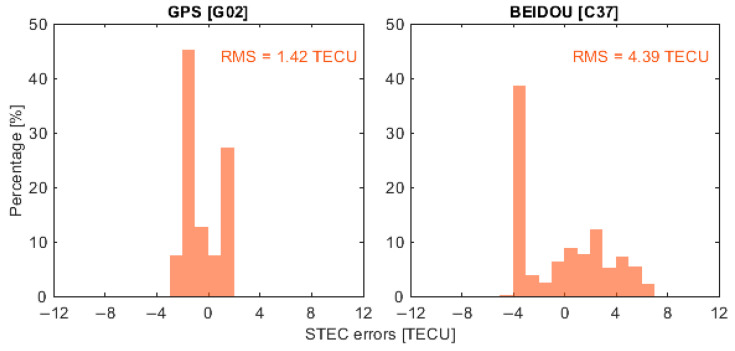
Slant ionospheric error distribution of selected satellites with PPP-B2b products in static scenarios.

**Figure 8:**
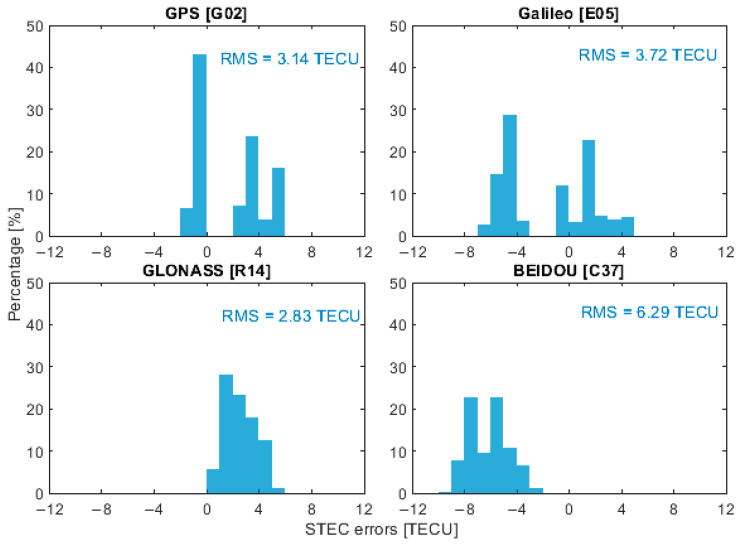
Slant ionospheric error distribution of selected satellites with CNES products in kinematic scenarios.

**Figure 9:**
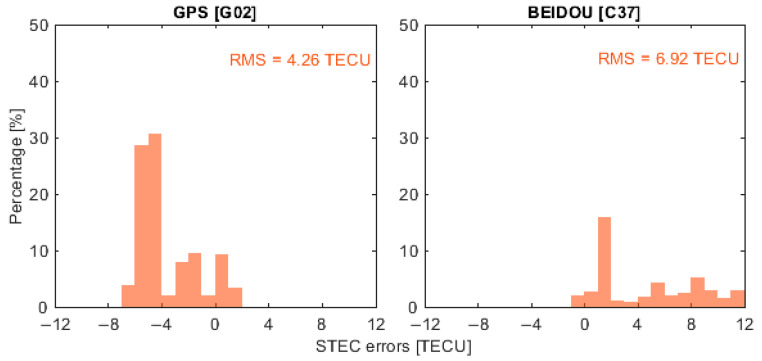
Slant ionospheric error distribution of selected satellites with PPP-B2b products in kinematic scenarios.

To represent the performance of ionospheric corrections from different GNSS constellations, four satellites, G02, E05, R14 and C37, were selected as representative examples, corresponding to GPS, Galileo, GLONASS and BeiDou, respectively. It is important to note that the BeiDou PPP-B2b service currently provides real-time correction products only for BeiDou and GPS satellites. Therefore, when using PPP-B2b products, the ionospheric delay analysis depicted in the following was limited to satellites G02 and C37. Figure 6, Figure 7, Figure 8 and Figure 9 present the error distributions of slant ionospheric delays, expressed in terms of STEC errors, estimated by the smartphone and the Trimble NetR9 receiver under different real-time product configurations, including CNES and BeiDou PPP-B2b products, for both static and kinematic cases.

In the static scenario, ionospheric corrections derived from the smartphone demonstrated consistent temporal behavior with those estimated by the geodetic-grade Trimble NetR9 receiver across all selected satellites. The discrepancies were confined to a limited range, demonstrating that the smartphone could reliably capture the overall ionospheric variation patterns, while exhibiting slightly higher variability than the geodetic-grade reference receiver. Quantitatively, the root mean square (RMS) errors of the ionospheric corrections for satellites G02, E05, R14, and C37 were 2.57, 3.33, 1.14, and 2.12 total electron content unit (TECU), respectively, with corresponding circular error probable at 68% (CEP68) values of 2.78, 3.66, 1.23, and 2.40 TECU. When using the BeiDou PPP-B2b real-time correction products that are available only for GPS and BeiDou, the RMS errors for G02 and C37 were 1.42 and 4.39 TECU, and the CEP68 values were 1.55 and 4.79 TECU, respectively.

In the kinematic scenario, the accuracy of smartphone-derived ionospheric corrections was approximately 1 m, corresponding to around 6 TECU. While this accuracy is lower than that achieved by the Trimble NetR9 receiver, the discrepancy between the two solutions remained within the meter level. The RMS errors for G02, E05, R14, and C37 were 3.14, 3.72, 2.83, and 6.29 TECU, respectively, with corresponding CEP68 values of 3.45, 4.17, 3.25, and 6.94 TECU. When using the BeiDou PPP-B2b products, the RMS errors for G02 and C37 increased to 4.26 and 6.92 TECU, with CEP68 values of 4.99 and 7.61 TECU, respectively. These results suggest that, although less precise than geodetic-grade solutions, smartphones can still provide meaningful ionospheric correction information under both static and dynamic conditions.

Table 2 presents a comprehensive summary of the ionospheric correction errors for all available satellites under both static and kinematic scenarios. The average RMS values for the static and kinematic scenarios are 2.78 TECU (CNES), 3.87 TECU (PPP-B2b) and 4.35 TECU (CNES), 5.93 (PPP-B2b), which are consistent with the results discussed above, further reinforcing the conclusion that smartphone-derived ionospheric corrections exhibit good agreement with geodetic-grade estimates, particularly under static conditions, and maintain meter-level accuracy under kinematic conditions.

**Figure 10:**
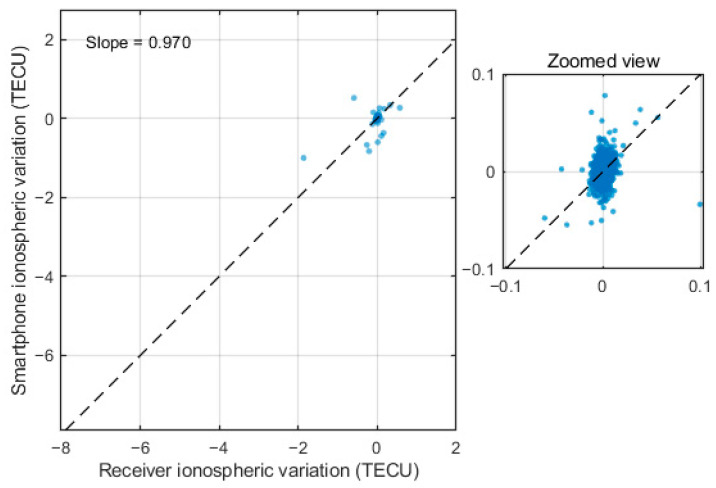
Similarity of slant ionospheric variations with the smartphone and receiver. The dashed line indicates the unity reference line (slope = 1), and the blue dots represent the first-differenced ionospheric observations from the smartphone and the geodetic receiver.

A scatterplot comparing the smartphone and receiver ionospheric series is shown in Figure 10. We present a scatterplot of the first-differenced series to emphasize the similarity in temporal variations, which shows a strong correlation and a near-unity slope of 0.970. This indicates that the smartphone captures ionospheric variation amplitudes that are highly consistent with those observed by the geodetic receiver. The agreement further confirms that the smartphone measurements preserve the underlying ionospheric dynamics despite their higher observation noise.

The application of different real-time precise products reveals discernible variations in performance; however, both satellite-based (PPP-B2b) and ground-based (CNES) services demonstrate the capacity to deliver accurate ionospheric corrections under their respective operational frameworks. The ionospheric corrections derived from ground-based products benefit from dense tracking networks and multi-constellation support, which facilitate accurate satellite clock estimation and stable separation of ionospheric parameters. Meanwhile, satellite-based real-time products provide globally accessible corrections through broadcast services and exhibit robust and consistent performance in regions with effective service coverage. Although their supported constellations differ from those of ground-based networks, PPP-B2b products are well aligned with the requirements of large-scale real-time positioning applications, particularly for mass-market and wide-area use cases.

Despite the increased noise level in smartphone observations, the ionospheric corrections estimated from smartphones with different real-time products remain within the meter level relative to the reference receiver. This can be explained by the fact that ionospheric delays are slowly varying parameters dominated by large-scale spatial structures, making them less sensitive to high-frequency observation noise. Consequently, even with noisier pseudorange and carrier-phase measurements, smartphones are still capable of capturing the main ionospheric behavior.

### 3.3. Positioning Performance Analysis with Smartphone-Based Ionospheric Corrections

Based on the evaluated ionospheric corrections, their impact on user-side positioning performance was further analyzed. This assessment focused exclusively on the kinematic scenario, and the positioning accuracy was evaluated under different ionospheric augmentation strategies, including corrections generated from the base smartphone and those derived from the Trimble NetR9 receiver, using various real-time precise products.

**Figure 11:**
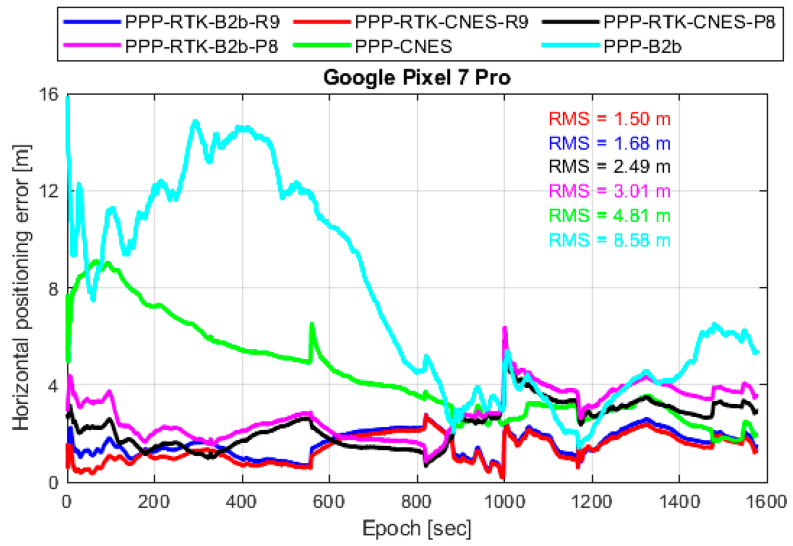
Horizontal positioning performance of the Google Pixel 7 Pro augmented by ionospheric corrections derived from a Google Pixel 8 and a geodetic receiver using CNES and PPP-B2b products.

Figure 11 illustrates the positioning performance of the user smartphone under ionospheric-augmented PPP modes from the geodetic receiver and the smartphone with different real-time products. The results demonstrate that the positioning performance of the user smartphone with ionospheric corrections broadcast from the base smartphone is significantly improved compared with the conventional PPP solution, which is 74.7% and 54.9% for the CNES and PPP-B2b products, respectively. The horizontal positioning errors are notably reduced, and the convergence time is substantially shortened.

This horizontal positioning improvement is mainly attributed to the additional ionospheric constraints introduced into the PPP model. By providing external ionospheric information, the strong correlation between ionospheric delay parameters and carrier-phase ambiguities is effectively mitigated, which is particularly critical under the high noise conditions of smartphone observations, allowing faster convergence and improved ionospheric parameters. Furthermore, a comparison between ionospheric corrections generated from the smartphone and those obtained from the Trimble NetR9 receiver reveals that the positioning performance of the user smartphone becomes comparable after convergence.

Although the ionospheric corrections derived from the smartphone are noisier, their accuracy is sufficient to constrain the ionospheric parameters within reasonable bounds, thereby preventing error absorption into other parameters such as ambiguities and tropospheric delays. These results show that, although centimeter-level ionospheric accuracy is required for applications targeting centimeter-level positioning, meter-level ionospheric corrections are already sufficient to significantly improve user-side PPP performance relative to the conventional PPP solution. In particular, such corrections provide effective constraints during the convergence stage, leading to faster convergence, while the positioning performance after convergence remains comparable.

## 4. Conclusions

This study evaluated the feasibility and performance of estimating slant ionospheric corrections from smartphones and applying them within a PPP-RTK framework. A real-world field experiment was conducted using Android smartphones together with a geodetic-grade Trimble NetR9 receiver under both static and kinematic scenarios. The results demonstrate that, under open-sky conditions, although smartphone-derived ionospheric corrections exhibit higher noise levels, they are able to reliably capture the overall ionospheric variation patterns observed by geodetic-grade references and maintain sufficient accuracy to support practical ionospheric augmentation.

Quantitative analyses showed that meter-level ionospheric corrections from smartphones are effective in constraining ionospheric parameters during PPP processing based on the open-sky experimental data analyzed in this study, especially during the convergence phase. This capability prevents significant error absorption into ambiguities and tropospheric delays and leads to improved user-side positioning performance. While the corrections do not reach geodetic-grade precision, they demonstrate a substantial improvement in positioning accuracy compared with conventional PPP solutions under open-sky dynamic conditions.

A comparative investigation of different real-time precise product sources demonstrates that, within the open-sky scenarios considered in this work, both ground-based and satellite-based augmentation services can effectively support smartphone-based ionospheric delay estimation within their respective operational frameworks. The results confirm the feasibility of deriving meaningful ionospheric information from low-cost smartphone GNSS observations, despite their high noise characteristics. Future work will explore the utilization of crowdsourced smartphone GNSS data to enhance the availability and spatial coverage of wide-area ionospheric corrections for mass-market applications.

## Figures and Tables

**Table 1:** Configuration of ionospheric corrections estimated from the smartphone.

Elevation angle threshold	7.0°
Signal-to-noise ratio threshold	15.0dB−Hz
Ephemeris	CNES/PPP-B2b CNES/PPP-B2b Pseudorange + carrier phase
Pseudorange hardware bias	
Observation types used	
Constellations	GPS + Galileo + GLONASS + BeiDou (CNES); GPS + BeiDou (PPP-B2b)
Slant ionospheric delay	Estimated using random walk model with process noise of $1\times 10^{-6}$ m
Ionospheric constrain option	Ionospheric corrections estimated from the smartphone
Tropospheric wet delay	Hydrostatic part: corrected using Saastamoinen model with GPT meteorological model and global mapping function (GMF)Wet part: estimated using random walk model with process noise of $1\times 10^{-6}$ m and GMF
Estimator	Kalman filter
Measurement noise model	C/N0-dependent model $\sigma_{P}=5+10^{4}\times 10^{-\frac{C/N_{0}}{10}}$ m $\sigma_{L}=0.01+50\times 10^{-\frac{C/N_{0}}{10}}$ m
Coordinate process noise	30.0 m

**Table 2:** Statistical results of ionospheric correction error for all available satellites (unit: TECU).

Mode	Products	System	RMS	STD	CEP68	CEP95
Static	CNES	GPS	2.34	2.04	2.78	4.05
		Galileo	3.12	2.97	3.66	5.62
		GLONASS	2.87	1.65	3.13	4.94
		BeiDou	2.79	2.31	2.96	5.05
	PPP-B2b	GPS	3.19	1.53	3.29	5.58
		BeiDou	4.55	3.59	4.79	8.14
Kinematic	CNES	GPS	2.51	2.57	2.71	4.34
		Galileo	4.29	3.33	4.50	7.55
		GLONASS	3.74	2.14	3.89	6.66
		BeiDou	6.86	3.13	6.94	12.49
	PPP-B2b	GPS	4.54	2.20	4.99	8.04
		BeiDou	7.31	4.39	7.61	13.38

## Data Availability

The GNSS dataset associated with this study is publicly accessible in the GitHub repository (https://github.com/zyejia524605-ui/data_sensors, accessed on 21 January 2026).
